# Application of the horse grimace scale in horses with dental disease: Preliminary findings

**DOI:** 10.1002/vetr.4800

**Published:** 2024-11-09

**Authors:** Amelia E. Sidwell, Marco Duz, Bradley Hill, Sarah Freeman, Sam L. Hole

**Affiliations:** ^1^ School of Veterinary Medicine and Science, University of Nottingham Sutton Bonington UK; ^2^ Pool House Equine Hospital Fradley UK

**Keywords:** dental pain, grimace scale, equine dentistry

## Abstract

**Background:**

Dental disease is a common but often under‐recognised condition in horses, possibly due to an inability to recognise clinical signs of oral discomfort. Some dental disorders are reportedly more painful than others, but there is no current metric by which dental pain can be objectively assessed. This study aimed to determine whether a facial expression‐based pain scale offered an objective and reliable method for assessing dental pain in horses. It was hypothesised that dental disorders affecting the periodontium would produce high pain scores.

**Methods:**

Twelve horses with dental disease were evaluated for pain using a numerical rating scale (NRS) and a horse grimace scale (HGS) by blinded observers using still, lateral photographs.

**Results:**

Interobserver reliability was poor across all observers when both the NRS (intraclass correlation coefficient [ICC] = 0.36) and the HGS (ICC = 0.27) were used in horses with dental disease. The highest mean scores were given for horses with equine odontoclastic tooth resorption and hypercementosis (EOTRH) and periodontal disease (PD).

**Limitations:**

This study has a small sample size of both horses and questionnaire respondents, and the respondent demographics are not representative of the wider veterinary population Furthermore, no positive or negative controls were used for the pain scoring.

**Conclusions:**

The results of this study indicate the unreliability of tools designed for identifying acute pain for assessing chronic pain, such as dental pain. A more dental‐specific ethogram is required to accurately identify dental pain in horses. Both the NRS and HGS produced the highest mean scores for EOTRH and PD, supporting existing literature that these conditions are associated with more obvious signs of pain.

## INTRODUCTION

Dental disease is one of the most common chronic healthcare conditions in domesticated animals, including horses.^1^, ^2^, ^3^ However, its presence is often missed, possibly as it is less visible than other chronic health conditions, such as orthopaedic disorders.

Clinical signs of dental disease can be difficult for horse owners to identify. Quidding, halitosis and external facial swellings are associated with dental disease in horses but are not present in many cases.^4^, ^5^ Some clinical signs may go unrecognised for many months before diagnosis, with horses often reported as ‘asymptomatic’, and significant dental pathology diagnosed only during routine dental assessments.^6^, ^7^ Dental disease can result in changes in both general and ridden behaviour, although these changes can be subtle in nature, are not specific to dental disease,^8^, ^9^ and may only be recognised by owners retrospectively.^6^ Therefore, the diagnosis and treatment of dental disease often relies on regular, routine oral examinations by a veterinarian or dental technician.

Owners have confidence in their veterinarian’s ability to accurately recognise pain, although many equine veterinarians consider their ability to recognise and quantify pain to be insufficient.^11^, ^12^, ^13^

Behavioural changes serve as reliable indicators of pain or discomfort in horses,^14^ and the same is likely true for oral pain.^10^ However, the evolutionary advantage conferred by prey animals displaying minimal signs of pain may contribute to more subtle changes in behaviour in horses experiencing pain or an absence of such behaviours in the presence of a predatory species such as humans.^15^ Behavioural changes may therefore be easily missed by owners and clinicians alike.^16^, ^17^

Changes in facial expression are considered the most consistent expression of pain in people, often revealing that a person is experiencing pain even when asked to conceal it. Pain in people is characterised by lowering of the eyebrows, narrowing of the eyes, wrinkling of the nose and raising of the upper lip.^18^ Similarly, pain‐related facial action units (FAUs) have been identified in other species, including horses and donkeys,^19^, ^20^, ^21^ resulting in the development of various grimace scales.^22^, ^23^, ^24^ These pain coding systems have proven to be valuable tools for assessing pain in clinical settings, being quick to perform and with good interobserver reliability, even in operators with little training.^25^

Grimace scales and facial expressions have been used to detect orofacial pain in a number of veterinary species, and various facial coding systems have been developed for human use, including for the detection of pain in non‐verbal or cognitively impaired individuals.^26^, ^27^, ^28^, ^29^ The horse grimace scale (HGS) has undergone further validation and has been used to assess oral health and dental disease in horses.^30^, ^31^ Dental disorders involving the periodontium, such as equine periodontal disease (PD) and equine odontoclastic tooth resorption and hypercementosis (EOTRH), are reported as being extremely painful conditions, with the potential to negatively impact quality of life.^32^, ^33^ However, there are currently no existing studies objectively comparing pain associated with different dental disorders in horses.

The first objective of this pilot study was to recruit hospitalised horses with a range of dental pathologies and compare the inter‐ and intraobserver variability of the HGS with that of a numerical rating scale (NRS) when used by blinded individuals with varying levels of clinical experience. It was hypothesised that the HGS would offer better inter‐ and intraobserver reliability than an NRS, regardless of operator experience, and could therefore improve objectivity when assessing pain in dental patients.

The second objective was to compare pain scores between individual horses to determine any possible correlation between specific dental disorders and pain scales. An improved understanding of which dental disorders are associated with higher pain scores could help clinicians target treatment towards dental pathology more likely to be responsible for pain, particularly where multiple lesions are identified.

## MATERIALS AND METHODS

### Animal selection

Twelve horses admitted to a UK referral hospital for exodontia between August 2022 and January 2023 were selected for inclusion in this study. These horses had no known comorbidities and no history of analgesic use within the previous 2 weeks. Horses not accustomed to being stabled or with a history of stereotypical behaviour were excluded from the study for observer safety and due to the potential impact of other negative emotional states on HGS scores.^34^ As the HGS has undergone some previous validation in horses with dental disease^30^ and the objective of this study was to compare the reliability of the HGS between observers, the decision was taken not to include a control population of horses without dental disease.

### Dental disease

All dental diagnoses were made or confirmed (for referred cases) by a specialist in equine dentistry (EVDC diplomate) based on a detailed oral examination, including oroscopy (Storz Telepak) and radiography. Horses were subsequently divided into categories on the basis of their diagnosis.

### Pain scoring in situ

Horses were evaluated prior to any treatment being undertaken (all diagnostics had been performed on a previous occasion). After admission, the horses were placed in a quiet stable for a minimum of 30 minutes to acclimatise. Haynets and a grille that had been placed over the door to minimise external distractions were removed while the horses were assessed, as both had been shown to interfere with the ability to accurately observe horses during initial methodology trials. This was deemed sufficient for most horses to settle into a normal resting cycle.^14^

A single observer then entered the stable and remained present for a 10‐minute period of quiet observation, during which pain scoring was conducted using an NRS, the equine Utrecht University facial assessment of pain (EQUUS‐FAP) tool^35^ and the HGS.^23^ All horses were assessed in situ by the same researcher throughout the study (Master's research student and resident in equine dentistry, AS). The researcher was not blinded to the diagnoses. For the NRS, horses were scored between 0 (no pain) and 5 (severe pain). For the HGS, six FAUs were assigned a score of 0 (not present), 1 (moderately present) or 2 (obviously present), resulting in a maximum possible score of 12. Similarly, individual parameters were given a score between 0 and 2 using the EQUUS‐FAP, with a maximum possible score of 24.

The EQUUS‐FAP has been shown to have poor interobserver reliability in horses with chronic pain, including dental disorders,^36^ and pilot observations in the development of the EQUUS‐FAP suggested that remote assessment by video footage was inadequate for using the scale.^21^ The HGS can be performed on still images, lending itself more readily to blinded evaluation by remote observers^30^, ^37^; hence, only this scale was included in the questionnaire in the latter part of the study.

### Obtaining images

At the end of the observation period, multiple still, lateral photographs of the animal in a resting position, which was representative of its appearance during the observation period, were obtained using a 12‐megapixel camera (iPhone XR iOS 16.1.1, Apple) in a well‐lit area, at a distance of approximately 1 m from the animal. One image was selected at random by a blinded individual not involved with the study, to minimise bias.

### Questionnaire development

Photographs were compiled into a questionnaire using Jisc (www.jisc.ac.uk). The respondents were asked to evaluate the images and provide a score using an NRS ranging from 0 (no pain) to 5 (severe pain). Pain scoring was immediately repeated on the same images using the HGS. All respondents were provided with a chart demonstrating how to score each FAU. The respondents were blinded to the diagnoses of the horses.

The questionnaire was distributed via email to equine veterinarians, registered equine veterinary nurses (REVNs) and final‐year veterinary students (equine track). Five first‐opinion equine practices and advanced practitioners in equine dentistry known to the primary researcher were contacted, in addition to University of Nottingham clinicians and students and both the referral and first‐opinion clinicians of the practice in which the study was conducted. Purposive sampling was chosen to ensure respondents represented a range of different levels of clinical experience. The respondents were also asked to indicate what percentage of their caseload consisted of dentistry (less than 10%, 10%‒25%, 25%‒50% or over 50%) and rate their confidence in assessing dental pain (‘not confident’, ‘somewhat confident’ or ‘very confident’). The respondents were not asked to specify whether they had prior experience using the HGS.

### Data analysis

Questionnaire data were extracted from JISC, organised in an Excel spreadsheet and then imported into R (Rv4.2.1, R core team 2022) for further analysis. The six‐point NRS (0–5) was converted by multiplying each value by a factor of 2.4 to permit direct comparison with the HGS scores. The respondents were categorised into the following groups for data analysis: veterinarians qualified for 10 years or less, veterinarians qualified for over 10 years, veterinary students and veterinary nurses. Further data analysis was undertaken for veterinarians, comparing specialists and first‐opinion practitioners.

The variation around the mean was calculated and used to determine the coefficient of variation (CoV). The Wilcoxon‐signed rank test was used to compare the CoV between scales, including all responses for each horse and for individual groups.

Mixed‐effect logistic regression was used to compare the scores given to each horse by each group on each scale. The explanatory fixed effect variable was ‘group’, and the horse was used as the predefined random effect. Whether groups had a statistically significant difference was determined by deriving *p*‐values (significance set for *p* < 0.05) using Satterthwaite approximations.^38^

Interobserver reliability was assessed using intraclass correlation coefficients (ICCs). This analysis was performed using Excel (Microsoft).

## RESULTS

### Respondent characteristics

A total of 31 respondents (22 veterinarians, four veterinary students and five REVNs) completed the questionnaire, with an estimated response rate of 15%. Of the 22 veterinarians, 10 (45.5%) estimated their dentistry caseload to be less than 10%, eight (36.4%) estimated their dentistry caseload to be 10%‒25%, a single respondent (0.05%) estimated that they had a dentistry caseload of 25%‒50%, and three (0.14%) estimated they had a dentistry caseload of more than 50%. Of the respondents with a dentistry caseload of less than 10% (*n* = 10), seven of these (70%) were specialists in fields other than equine dentistry. Twenty‐three respondents (74.2%) self‐reported as ‘somewhat confident’ in their ability to assess dental pain, six described themselves as ‘not confident’ (19.4%) and only two (6.5%) described themselves as ‘very confident’. The two respondents who reported being ‘very confident’ in assessing dental pain were veterinarians with over 10 years of clinical experience and a large dental caseload (over 50%) or a further qualification in equine dentistry.

### Dental disease

The cases included cheek tooth fracture (*n* = 5), periapical infection (non‐fractured cheek tooth) (*n* = 3), PD (*n* = 1), EOTRH (*n* = 1) and developmental disorders (*n* = 2).

### Interobserver reliability

There was no significant difference in the CoV in the scores between first‐opinion veterinarians and specialists (*p* = 0.8), specialists and other (defined as veterinary students and REVNs) (*p* = 0.7) and first‐opinion veterinarians and other (*p* = 0.7) for either scale. This means that members of each of these groups tended to score around the mean.

There was a statistically significant difference in the NRS scores between veterinarians qualified for more than 10 years and those qualified for less than 10 years (*p* < 0.001), with veterinarians qualified for less than 10 years tending to give higher subjective scores than those qualified for more than 10 years. There was no significant difference between specialists and first‐opinion veterinarians (*p* = 0.1).

There was no significant effect of experience (expressed as years from graduation) on the HGS. There was a statistically significant difference in NRS scores between those who had graduated more than 10 years ago (reference) and those who had graduated 1‒2 years (*p* = 0.002) or 5‒10 years (*p* < 0.001) ago. However, there was no difference between those who had graduated more than 10 years ago and those who had graduated 3‒5 years ago (*p* = 0.2).

The ICC for all respondents was 0.36 for the NRS and 0.26 for the HGS, indicating poor reliability for both scales (<0.50) across all observers.^39^ The reliability between the NRS and the HGS was poor for non‐specialists, but both the NRS and HGS performed similarly (NRS = 0.36 and HGS = 0.36). When comparing the ICC for specialists, the ICC was 0.35 using the NRS and 0.19 using the HGS, suggesting that the HGS was even less reliable than the NRS in this particular group.

### Comparison of scores and dental pathology

The mean scores for each condition according to the HGS and NRS are listed in Table 1. EOTRH resulted in the highest mean pain score using both the NRS (3.38/5) and the HGS (7.56/12), followed by PD (2.70/5 for the NRS and 6.65/12 for the HGS).

**TABLE 1:** Mean observer scores for different dental disorders using the horse grimace scale (HGS) and a numerical rating scale (NRS)

Dental pathology	HGS	NRS
Tooth fracture (*n* = 5)	5.50	2.03
Periapical infection (*n* = 3)	5.03	2.35
Periodontal disease (*n* = 1)	6.65	2.70
EOTRH (*n* = 1)	6.80	3.38
Developmental disorders (*n* = 2)	3.98	1.51

*Note*: The maximum total scores for the HGS and NRS are 12 and 5, respectively.

Abbreviation: EOTRH, equine odontoclastic tooth resorption and hypercementosis.

## DISCUSSION

Various tools have been developed for assessing pain in horses,^25^ but many of these require further validation to determine their reliability in different pain states before they can be accepted for use in clinical practice. Despite the prevalence of dental disease in horses, many cases still go unrecognised, possibly because these horses do not display the ‘typical’ clinical signs associated with dental pathology.^4^, ^5^, ^6^, ^7^

The results of this study indicate that respondents in each group tended to score closely around the mean, regardless of whether the NRS or the HGS was used. These findings are similar to previous studies, where NRSs were able to discriminate well between extremes.^40^ However, unlike previous studies, the results of this study demonstrate poor reliability between observers using both the NRS and the HGS. ICC was poorer for the HGS compared to the NGS in all groups. Blinded observers in previous studies using the HGS have been trained in its use.^30^, ^37^ One possible explanation for the poorer ICC reported in this study may be that the untrained observers used the HGS as an NRS rather than assessing each FAU separately, resulting in a greater score variation than with a smaller (0–5) scale. The results were not analysed to determine whether interobserver reliability improved towards the end of the questionnaire as respondents became more familiar with the scale. Training in the use of the HGS may have improved the consistency of scores, although the amount of training deemed sufficient to accurately apply the HGS is still unknown, and may be variable dependent on the individual and their familiarity with horses.^23^, ^37^, ^41^, ^42^

A previous study undertaken by Coneglian et al.^30^ demonstrated reduced HGS scores in horses undergoing routine odontoplasty, with significant agreement between four observers based on still photographs. It would perhaps be expected that horses with severe dental disease necessitating exodontia would show greater outward signs of discomfort than those undergoing routine odontoplasty, resulting in more obvious alterations in facial expression. This study, which used a greater number of observers of different levels of clinical experience, contradicted these earlier findings. This further highlights the challenges in accurately and objectively assessing dental pain in horses. It may be that horses with dental pathology do not display facial expressions of pain that are recognisable to clinicians, particularly in the presence of an observer, or that clinicians lack the skills to accurately evaluate FAUs without further training, particularly as these could be mistaken for other negative emotional states.^34^, ^43^

There was a trend towards lower scores using the NRS in veterinarians with more than 10 years of experience. This may suggest that a process of desensitisation occurs in veterinary professionals when repeatedly exposed to pain and suffering, as has been demonstrated in human healthcare providers.^44^, ^45^ Veterinarians with board certification are more likely to provide lower pain scores for various common disorders and surgical procedures in horses than those without board certification.^11^ This is perhaps unsurprising, as those working at the referral level are likely to have greater exposure to patients with more painful conditions, such as colic, or surgical cases. However, the results of this study did not show any significant difference between specialists and first‐opinion practitioners.

Seventy‐four percent of respondents were ‘somewhat confident’ in their ability to recognise dental pain in horses, which is similar to the results of prior studies in the veterinary literature.^11^, ^13^, ^46^ While not investigated as part of this study, an individual's self‐confidence in recognising pain may not necessarily influence pain scores or the provision of analgesia.^47^

### Limitations

One significant limitation was the small sample size of 12 horses and the limited number of respondents. Additionally, there was a significant bias towards veterinarians working at the referral level, so the results may not reflect the breadth of the profession. However, there was a relatively equal number of specialists (*n* = 10) and first‐opinion practitioners (*n* = 12), permitting direct comparison between groups.

Additionally, while those completing the questionnaire were blinded to the exact diagnosis, they were aware that the questionnaire aimed to evaluate pain in horses with dental disease. As such, it is possible that scores were influenced by the participants’ perceptions of dental pain compared with other conditions.

The in situ scorer was aware of the exact diagnosis of the horses included in the study. It is possible that preconceived notions regarding the degree of pain associated with different dental disorders could result in bias.^11^, ^47^ While there was no significant difference between in situ HGS scores and the average HGS scores given by blinded observers, given the poor overall reliability of the HGS in this study, it cannot be concluded that using the HGS helps overcome such biases.

No controls were used in this study, as the HGS has undergone previous validation in horses with dental disease^30^ and the primary goals of this study were to assess the interobserver reliability of the scale and compare scores across different dental disorders. However, it must be acknowledged that the participants in this study were untrained in using the HGS. As such, the inclusion of control animals without dental disease may have been valuable.

Although horses included in study had no known comorbidities, one significant limitation of grimace scales is that it they are not pain specific.^23^, ^30^, ^37^ It may be that more dental‐specific indicators of pain are needed to accurately evaluate dental patients. A dynamic scoring system may improve interobserver reliability and eliminate potential bias associated with obtaining and evaluating still images.^41^ Yawning and teeth grinding were frequently observed by the in situ observer. These behaviours are associated with changes in sympathetic tone and have been included in other pain‐scoring systems to monitor acute, head‐related pain.^21^ However, the same scale is less reliable for the assessment of chronic pain, including dental disease.^36^

Determining whether certain behaviours are more frequently observed in horses with dental pain compared to other painful conditions (positive controls) is also needed, as their inclusion could help improve the sensitivity of pain scales in horses with dental disease.

The presence of an observer may impact certain behaviours.^15^ Remote assessment of video footage would possibly permit more accurate assessment and cataloguing of behaviours seen in horses with dental disease. However, not all clinical settings provide the opportunity for remote assessment, particularly in an ambulatory environment. As the authors feel it is important that tools designed for evaluating pain are validated in a setting reflecting their intended use, the decision was taken to obtain images in situ rather than via remote assessment.

## CONCLUSIONS

EOTRH resulted in the highest mean pain score using both scales, followed by PD. EOTRH is a progressive condition in horses, with the resulting development of painful PD negatively impacting quality of life.^48^, ^49^, ^50^ While the reliability between observers was poor for both scales, a trend towards higher scores for the horses with these conditions may support the existing literature that conditions affecting the periodontium are among the most painful dental disorders in horses.^51^ However, only a small number of horses were included in the study, with a bias towards horses with dental fractures. Further studies on a larger population of horses using a more reliable method for assessing pain will add further information on this issue.

The results of the questionnaire indicate that veterinarians are generally confident in their ability to recognise dental pain in horses. However, the poor interobserver reliability of both the NRS and the HGS suggests that veterinarians lack the tools with which to objectively measure pain and that there is little agreement between different veterinary professionals. Furthermore, there appears to be the possibility of desensitisation to pain with increasing experience. Veterinarians should be mindful of this.

Overall, this study indicates that the HGS may not be an appropriate tool for objectively detecting or measuring dental pain in horses by untrained observers. Therefore, further work is needed to better understand the behaviours of horses with dental disease and develop tools for evaluating dental pain in a clinical setting.

## AUTHOR CONTRIBUTIONS

Amelia E. Sidwell conceived the original idea and study design, collected data and wrote initial drafts of the paper. Brad Hill provided input into the original concept, study design and final paper reviews. Marco Duz ensured that the necessary ethical approval had been obtained and performed all the data analysis. Sam L. Hole facilitated initial data collection from hospitalised patients. Sarah Freeman provided invaluable input regarding manuscript structure and publication. All the authors were involved in manuscript writing.

## CONFLICT OF INTEREST STATEMENT

The authors declare they have no conflicts of interest.

## ETHICS STATEMENT

Ethical approval was granted by the approval of the Committee for Animals Research and Ethics of the School of Veterinary Medicine and Science at the University of Nottingham. Informed consent was obtained from the horse owners.

## Data Availability

All data are available from the authors upon request.

## References

[1] O'Neill DG , Church DB , McGreevy PD , Thomson PC , Brodbelt DC . Prevalence of disorders recorded in cats attending primary‐care veterinary practices in England. Vet J. 2014;202(2):286–291. 25178688 10.1016/j.tvjl.2014.08.004

[2] Brigham EJ , Duncanson GR . Case study of 100 horses presented to an equine dental technician in the UK. Equine Vet Educ. 2000;12(2):63–67.

[3] Dixon PM , Dacre I . A review of equine dental disorders. Vet J. 2005;169(2):165–187. 15727909 10.1016/j.tvjl.2004.03.022

[4] Dixon PM , Tremaine WH , Pickles K , Kuhns L , Hawe C , McCann J , et al. Equine dental disease. Part 3: A long‐term study of 400 cases: disorders of wear, traumatic damage and idiopathic fractures, tumours and miscellaneous disorders of the cheek teeth. Equine Vet J. 2000;32(1):9–18. 10661379 10.2746/042516400777612099

[5] Rowley KJ , Townsend NB , Chang YR , Fiske‐Jackson AR . A computed tomographic study of endodontic and apical changes in 81 equine cheek teeth with sagittal fractures. Equine Vet J. 2022;54(3):541–548. 34060137 10.1111/evj.13475

[6] Taylor L , Dixon PM . Equine idiopathic cheek teeth fractures. Part 2: A practice‐based survey of 147 affected horses in Britain and Ireland. Equine Vet J. 2007;39(4):322–326. 17722723 10.2746/042516407x182802

[7] Dixon PM , Kennedy R , Reardon RJM . Equine 'idiopathic' and infundibular caries‐related cheek teeth fractures: a long‐term study of 486 fractured teeth in 300 horses. Front Vet Sci. 2021;8:646870. 34124217 10.3389/fvets.2021.646870 PMC8192706

[8] von Borstel UK , Glißman C . Alternatives to conventional evaluation of rideability in horse performance tests: suitability of rein tension and behavioural parameters. PLoS ONE. 2014;9(1):e87285. 24489890 10.1371/journal.pone.0087285 PMC3906164

[9] Dyson S , Berger J , Ellis AD , Mullard J . Development of an ethogram for a pain scoring system in ridden horses and its application to determine the presence of musculoskeletal pain. J Vet Behav. 2018;23:47–57.

[10] Pehkonen J , Karma L , Raekallio M . Behavioral signs associated with equine periapical infection in cheek teeth. J Equine Vet Sci. 2019;77:144–150. 31133309 10.1016/j.jevs.2019.03.005

[11] Sellon DC , Sanz M , Kopper JJ , Mattei D . Pain severity scores for common equine disorders as provided by horse owners and equine veterinarians. Equine Vet J. 2022;54(6):1094–1102. 35034381 10.1111/evj.13559

[12] Hugonnard M , Leblond A , Keroack S , Cadoré J , Troncy E . Attitudes and concerns of French veterinarians towards pain and analgesia in dogs and cats. Vet Anaesth Analg. 2004;31(3):154–163. 15268686 10.1111/j.1467-2987.2004.00175.x

[13] Morales‐Vallecilla C , Ramírez N , Villar D , Díaz M , Bustamante S , Ferguson D . Survey of pain knowledge and analgesia in dogs and cats by Colombian veterinarians. Vet Sci. 2019;6(1):6. 30634671 10.3390/vetsci6010006 PMC6466334

[14] Torcivia C , McDonnell S . Equine discomfort ethogram. Animals. 2021;11(2):580. 33672338 10.3390/ani11020580 PMC7931104

[15] Torcivia C , McDonnell S . In‐person caretaker visits disrupt ongoing discomfort behavior in hospitalized equine orthopedic surgical patients. Animals. 2020;10(2):210. 32012670 10.3390/ani10020210 PMC7070845

[16] Cohen S , Beths T . Grimace scores: tools to support the identification of pain in mammals used in research. Animals. 2020;10(10):1726. 32977561 10.3390/ani10101726 PMC7598254

[17] Taylor PM , Pascoe PJ , Mama KR . Diagnosing and treating pain in the horse. Where are we today? Vet Clin North Am Equine Pract. 2002;18:1‒19. 12064173 10.1016/s0749-0739(02)00009-3

[18] Cohn JF , Ambadar Z , Ekman P . Observer‐based measurement of facial expression with the facial action coding system. In: Coan JA , Allen JJB , editors. Handbook of emotion elicitation and assessment. New York: Oxford University Press; 2007. p. 203–221.

[19] Orth EK , Navas González FJ , Iglesias Pastrana C , Berger JM , Jeune SSL , Davis EW , et al. Development of a donkey grimace scale to recognize pain in donkeys (*Equus asinus*) post castration. Animals. 2020;10(8):1411. 32823676 10.3390/ani10081411 PMC7459673

[20] Dalla Costa E , Minero M , Lebelt D , Stucke D , Canali E , Leach MC . Development of the horse grimace scale (HGS) as a pain assessment tool in horses undergoing routine castration. PLoS One. 2014;9(3):e92281. 24647606 10.1371/journal.pone.0092281 PMC3960217

[21] van Loon JPAM , Van Dierendonck MC . Monitoring equine head‐related pain with the equine Utrecht University scale for facial assessment of pain (EQUUS‐FAP). Vet J. 2017;220:88–90. 28190503 10.1016/j.tvjl.2017.01.006

[22] Evangelista MC , Monteiro BP , Steagall PV . Measurement properties of grimace scales for pain assessment in nonhuman mammals: a systematic review. Pain. 2022;163(6):e697–e714. 34510132 10.1097/j.pain.0000000000002474

[23] Langford DJ , Bailey AL , Chanda ML , Clarke SE , Drummond TE , Echols S , et al. Coding of facial expressions of pain in the laboratory mouse. Nat Methods. 2010;7(6):447–449. 20453868 10.1038/nmeth.1455

[24] Sotocina SG , Sorge RE , Zaloum A , Tuttle AH , Martin LJ , Wieskopf JS , et al. The rat grimace scale: a partially automated method for quantifying pain in the laboratory rat via facial expressions. Mol Pain. 2011;7:55. 21801409 10.1186/1744-8069-7-55 PMC3163602

[25] De Grauw JC , van Loon JPAM . Systematic pain assessment in horses. Vet J. 2016;209:14–22. 26831169 10.1016/j.tvjl.2015.07.030

[26] Mogil JS , Pang DSJ , Silva Dutra GG , Chambers CT . The development and use of facial grimace scales for pain measurement in animals. Neurosci Biobehav Rev. 2020;116:480–493. 32682741 10.1016/j.neubiorev.2020.07.013

[27] Sperry MM , Yu YH , Welch RL , Granquist EJ , Winkelstein BA . Grading facial expression is a sensitive means to detect grimace differences in orofacial pain in a rat model. Sci Rep. 2018;8(1):13894. 30224708 10.1038/s41598-018-32297-2 PMC6141616

[28] Watanabe R , Doodnaught GM , Evangelista MC , Monteiro BP , Ruel HLM , Steagall PV . Inter‐rater reliability of the feline grimace scale in cats undergoing dental extractions. Front Vet Sci. 2020;7:302. 32548134 10.3389/fvets.2020.00302 PMC7272704

[29] Rojo R , Prados‐Frutos JC , López‐Valverde A . Pain assessment using the facial action coding system. A systematic review. Med Clin Engl Ed. 2015;145(8):350–355. 10.1016/j.medcli.2014.08.010 25433779

[30] Coneglian MM , Duarte Borges T , Weber SH , Godoi Bertagnon H , Michelotto PV . Use of the horse grimace scale to identify and quantify pain due to dental disorders in horses. Appl Anim Behav Sci. 2020;225:104970.

[31] Coneglian MM , Weber SH , Michelotto Junior PV . Influence of oral health on the facial expressions and on the acupuncture examination in equines. Arq Bras Med Vet Zootec. 2023;75(3):415–424.

[32] Limone LE . Update on equine odontoclastic tooth resorption and hypercementosis. Vet Clin North Am Equine Pract. 2020;36(3):671–689. 33067098 10.1016/j.cveq.2020.08.006

[33] Kennedy RS , Dixon PM . The aetiopathogenesis of equine periodontal disease—a fresh perspective. Equine Vet Educ. 2018;30(3):161–168.

[34] Dalla Costa E , Bracci D , Dai F , Lebelt D , Minero M . Do different emotional states affect the horse grimace scale score? A pilot study. J Equine Vet Sci. 2017;54:114–117.

[35] Van Loon JPAM , Van Dierendonck MC . Monitoring acute equine visceral pain with the Equine Utrecht University Scale for Composite Pain Assessment (EQUUS‐COMPASS) and the Equine Utrecht University Scale for Facial Assessment of Pain (EQUUS‐FAP): a scale‐construction study. Vet J. 2015;206(3):356–364. 26526526 10.1016/j.tvjl.2015.08.023

[36] Van Loon JPAM , Macri L . Objective assessment of chronic pain in horses using the horse chronic pain scale (HCPS): a scale‐construction study. Animals. 2021;11(6):1826. 34207290 10.3390/ani11061826 PMC8234780

[37] Dalla Costa E , Stucke D , Dai F , Minero M , Leach M , Lebelt D . Using the horse grimace scale (HGS) to assess pain associated with acute laminitis in horses (*Equus caballus*). Animals. 2016;6(8):47. 27527224 10.3390/ani6080047 PMC4997272

[38] Luke SG . Evaluating significance in linear mixed‐effects models in R. Behav Res Methods. 2017;49(4):1494–1502. 27620283 10.3758/s13428-016-0809-y

[39] Koo TK , Li MY . A guideline for selecting and reporting intraclass correlation coefficients for reliability research. J Chiropr Med. 2016;15(2):155–163. 27330520 10.1016/j.jcm.2016.02.012 PMC4913118

[40] Sutton GA , Paltiel O , Soffer M , Turner D . Validation of two behaviour‐based pain scales for horses with acute colic. Vet J. 2013;197(3):646–650. 23993390 10.1016/j.tvjl.2013.04.007

[41] McKeown R . Validation of the horse grimace scale: exploring the effects of training and previous experience. Unitec Institute of Technology; New Zealand.

[42] Dai F , Leach M , MacRae AM , Minero M , Dalla Costa E . Does thirty‐minute standardised training improve the inter‐observer reliability of the horse grimace scale (HGS)? A case study. Animals. 2020;10(5):781. 32365927 10.3390/ani10050781 PMC7277819

[43] Anderson KA , Morrice‐West AV , Wong ASM , Walmsley EA , Fisher AD , Whitton RC , et al. Poor association between facial expression and mild lameness in thoroughbred trot‐up examinations. Animals. 2023;13(11):1727. 37889660 10.3390/ani13111727 PMC10251806

[44] Gregory NS , Harris AL , Robinson CR , Dougherty PM , Fuchs PN , Sluka KA . An overview of animal models of pain: disease models and outcome measures. J Pain. 2013;14(11):1255–1269. 24035349 10.1016/j.jpain.2013.06.008 PMC3818391

[45] Thomsen PT , Anneberg I , Herskin MS . Differences in attitudes of farmers and veterinarians towards pain in dairy cows. Vet J. 2012;194(1):94–97. 22516921 10.1016/j.tvjl.2012.02.025

[46] Beswick A , Dewey C , Johnson R , Dowsett‐Cooper J , Niel L . Survey of Ontario veterinarians’ knowledge and attitudes on pain in dogs and cats in 2012. Can Vet J. 2016;57:1274‒1280. 27928175 PMC5109631

[47] Dohoo SE , Dohoo IR . Factors influencing the postoperative use of analgesics in dogs and cats by Canadian veterinarians. Can Vet J. 1996;37:552‒556. 8877041 PMC1576376

[48] Lorello O , Foster DL , Levine DG , Boyle A , Engiles J , Orsini JA . Clinical treatment and prognosis of equine odontoclastic tooth resorption and hypercementosis. Equine Vet J. 2016;48(2):188–194. 25557855 10.1111/evj.12406

[49] Rahmani V , Häyrinen L , Kareinen I , Ruohoniemi M . History, clinical findings and outcome of horses with radiographical signs of equine odontoclastic tooth resorption and hypercementosis. Vet Rec. 2019;185(23):730. 31601733 10.1136/vr.105253 PMC7008772

[50] Hole SL , Staszyk C . Equine odontoclastic tooth resorption and hypercementosis. Equine Vet Educ. 2018;30(7):386–391. 10.1111/evj.12776 29067719

[51] Dixon PM , Tremaine WH , Pickles K , Kuhns L , Hawe C , McCann J , et al. Equine dental disease. Part 2: a long‐term study of 400 cases: disorders of development and eruption and variations in position of the cheek teeth. Equine Vet J. 1999;31(6):519–528. 10596936 10.1111/j.2042-3306.1999.tb03862.x

